# Abscopal Effects in Radio-Immunotherapy—Response Analysis of Metastatic Cancer Patients With Progressive Disease Under Anti-PD-1 Immune Checkpoint Inhibition

**DOI:** 10.3389/fphar.2019.00511

**Published:** 2019-05-14

**Authors:** Maike Trommer, Sin Yuin Yeo, Thorsten Persigehl, Anne Bunck, Holger Grüll, Max Schlaak, Sebastian Theurich, Michael von Bergwelt-Baildon, Janis Morgenthaler, Jan M. Herter, Eren Celik, Simone Marnitz, Christian Baues

**Affiliations:** ^1^Faculty of Medicine and University Hospital Cologne, Department of Radiation Oncology and Cyberknife Center, University of Cologne, Cologne, Germany; ^2^Faculty of Medicine and University Hospital Cologne, Radio Immune-Oncology Consortium, University of Cologne, Cologne, Germany; ^3^Faculty of Medicine and University Hospital Cologne, Center for Integrated Oncology (CIO Köln Bonn), University of Cologne, Cologne, Germany; ^4^Faculty of Medicine and University Hospital Cologne, Department of Diagnostic and Interventional Radiology, University of Cologne, Cologne, Germany; ^5^Department of Dermatology and Allergology, Ludwig-Maximilians University Munich, Munich, Germany; ^6^Department of Medicine III, University Hospital, Ludwig-Maximilians University Munich, Munich, Germany; ^7^Gene Center, Cancer- and Immunometabolism Research Group, Ludwig-Maximilians University Munich, Munich, Germany; ^8^Faculty of Medicine and University Hospital Cologne, Center for Molecular Medicine Cologne, University of Cologne, Cologne, Germany

**Keywords:** abscopal effect, PD-1, radio-immunotherapy, radiotherapy, combination treatment, advanced cancer disease, immune checkpoint inhibition

## Abstract

Immune checkpoint inhibition (ICI) targeting the programmed death receptor 1 (PD-1) has shown promising results in the fight against cancer. Systemic anti-tumor reactions due to radiation therapy (RT) can lead to regression of non-irradiated lesions (NiLs), termed “abscopal effect” (AbE). Combination of both treatments can enhance this effect. The aim of this study was to evaluate AbEs during anti-PD-1 therapy and irradiation. We screened 168 patients receiving pembrolizumab or nivolumab at our center. Inclusion criteria were start of RT within 1 month after the first or last application of pembrolizumab (2 mg/kg every 3 weeks) or nivolumab (3 mg/kg every 2 weeks) and at least one metastasis outside the irradiation field. We estimated the total dose during ICI for each patient using the linear quadratic (LQ) model expressed as 2 Gy equivalent dose (EQD2) using α/β of 10 Gy. Radiological images were required showing progression or no change in NiLs before and regression after completion of RT(s). Images must have been acquired at least 4 weeks after the onset of ICI or RT. The surface areas of the longest diameters of the short- and long-axes of NiLs were measured. One hundred twenty-six out of 168 (75%) patients received ICI and RT. Fifty-three percent (67/126) were treated simultaneously, and 24 of these (36%) were eligible for lesion analysis. AbE was observed in 29% (7/24). One to six lesions (mean = 3 ± 2) in each AbE patient were analyzed. Patients were diagnosed with malignant melanoma (MM) (*n* = 3), non-small cell lung cancer (NSCLC) (*n* = 3), and renal cell carcinoma (RCC) (*n* = 1). They were irradiated once (*n* = 1), twice (*n* = 2), or three times (*n* = 4) with an average total EQD2 of 120.0 ± 37.7 Gy. Eighty-two percent of RTs of AbE patients were applied with high single doses. MM patients received pembrolizumab, NSCLC, and RCC patients received nivolumab for an average duration of 45 ± 35 weeks. We demonstrate that 29% of the analyzed patients showed AbE. Strict inclusion criteria were applied to distinguish the effects of AbE from the systemic effect of ICI. Our data suggest the clinical existence of systemic effects of irradiation under ICI and could contribute to the development of a broader range of cancer treatments.

## Introduction

In addition to radiation therapy (RT), chemotherapy (CTX), and surgery, immunotherapy (IT) has been established as a fourth pillar of cancer treatment. Different treatment regimens and combination concepts are being evaluated and used in order to optimize treatment outcome of various tumor diseases.

RT is used for local treatment of malignant diseases. More than 50% of all patients with solid tumors are treated with RT only or in a combined treatment setting. The interaction of RT and patient's immune system has gained particular interest after the encouraging success of immune checkpoint inhibitors (ICIs) targeting the programmed death receptor 1 (PD-1) (Garon et al., 2015; Haanen and Robert, 2015; Robert et al., 2015; Ferris et al., 2016; Sharma et al., 2016; Younes et al., 2016; Long et al., 2017; Ok and Young, 2017). PD-1 checkpoint inhibitors act by suppression of an inhibitory T-cell pathway, namely the PD-1/PD-L1 axis. In metastatic malignant melanoma (MM), anti-PD1 therapy has been proven as superior treatment to chemotherapy as first-line therapy and after ipilimumab (anti-CTLA-4 antibody) failure (Ribas et al., 2015; Weber et al., 2015) and in non-small cell lung cancer (NSCLC) patients after progression to first-line chemotherapy (Vokes et al., 2018). Despite all advancements, not all patients benefit from treatment with ICIs, and different systemic therapies are less effective if the tumor does not contain a mutation that can be targeted. Looking for further treatment strategies, the combination of local irradiation, and ICIs led to promising results even beyond local tumor control (Kang et al., 2016; Salama et al., 2016). The mechanisms by which RT and IT synergistically modulate the immune response might also affect treatment-related side effects. Evidence shows that simultaneous administration of RT and ICIs as radio-immunotherapy (RIT) is considered safe and that the number of adverse events does not increase significantly (Bang et al., 2017; Hwang et al., 2018; Trommer-Nestler et al., 2018). The first report on an immune-mediated response to radiation therapy and the definition of the term “abscopal” in this context was published in 1953 describing the effects of ionizing radiation “at a distance from the irradiated volume but within the same organism” (Mole, 1953). The so-called abscopal effect (AbE) describes the regression of lesions or tumor or metastatic regions outside the radiation field induced by radiation.

Over time, there have been some reports of clinically observed AbEs, most commonly in highly immunogenic tumor entities (Abuodeh et al., 2016). The underlying mechanism of the AbE is still unclear. Most likely it is mediated by the activation of the immune system (Demaria et al., 2004) and is dependent on RT-induced cell damage leading to the release of cell fragments, neoantigens, cellular danger-associated molecular patterns (DAMPs), and cytokines (Formenti and Demaria, 2013). One way to improve the probability of the occurrence of AbEs through RT is to modulate the tumor microenvironment. This could be achieved by changing the radiation dose, fractionation, site of irradiation and timing, or by combined RT with other systemic therapies. The interactions of RT and IT might be able to immunize the patient against the tumor, acting like a type of “tumor vaccine” leading to a decrease of both tumor and metastases (Demaria and Formenti, 2009; Frey et al., 2012; Formenti and Demaria, 2013; Sharabi et al., 2015).

Currently, more and more case reports on the AbE are being published (Grimaldi et al., 2014; Chandra et al., 2015; Ribeiro Gomes et al., 2016). The incidence of AbEs is still rare and the radiation characteristics like fractionation, timing, fraction scheme, and total dose required for its occurrence remains unclear up until today. The actual occurrence of the AbE has not been well-evaluated in clinical studies so far. This retrospective single center study was conducted to evaluate AbEs in metastasized cancer patients treated with irradiation and simultaneous PD-1 inhibition with pembrolizumab or nivolumab.

## Materials and Methods

Out of a database of 168 patients treated with a PD-1 inhibitor between 2013 and 2017 at our center (University Hospital of Cologne) we retrospectively analyzed patients who received pembrolizumab or nivolumab and radiotherapy simultaneously. We included patients with any metastatic oncological disease with at least one not locally treated distant metastatic lesion outside V10% of the prescribed irradiation dose (volume of normal tissue receiving at least 10% dose).

The indication for RT was due to locally progressive disease under ICIs alone requiring symptomatic control. Disease progression was defined according to RECIST (Response Evaluation Criteria in Solid Tumors) version 1.1. Any irradiation concept with respect to fractionation scheme and irradiation dose like conventional radiation therapy (CFX), hypofractionated radiation therapy (HFX), stereotactic body radiation therapy (SBRT) or stereotactic radiosurgery (SRS), and multiple RT sessions during IT were permissible. Since patients could have received more than one RT at different sites and with different concepts we calculated the total irradiation dose during the IT period for each patient using the linear quadratic (LQ) model expressed as 2 Gy equivalent dose (EQD2) using an α/β value of 10 Gy, which has been assumed for tumors (Fowler, 1989; Stuschke and Pottgen, 2010).

Nivolumab was applied intravenously 3 mg/kg every 2 weeks, pembrolizumab 2 mg/kg every 3 weeks. Patients receiving any other systemic cancer treatment, such as ipilimumab, targeted therapy or chemotherapy during the IT or RT periods were excluded, while patients with previous use of systemic treatment were not excluded. We defined simultaneously applied radio-immunotherapy (RIT) as start of RT within 1 month after the first or last application of ICI.

AbE was defined as regression of lesions outside the irradiation field, more specifically outside the 10% iso-dose of the applied radiation dose. In order to distinguish AbE from the systemic effects of IT alone, radiological images were required to show progression or no change in non-irradiated lesion(s) during PD-1 inhibitor administration prior to RT application. If those lesions showed regression after one or more RTs, this was defined as AbE. Radiological images must have been acquired at least 4 weeks after the onset of ICI or RT for regression of lesions to be considered a reliable treatment effect. Patients and radiological images were regularly discussed in interdisciplinary panels.

All computed tomography (CT), magnetic resonance imaging (MRI), and/or positron emission tomography (PET) images were analyzed to identify lesions within and outside the irradiation field. The longest diameters of both the short-axis and long-axis of all non-irradiated lesions were measured and the resulting surface area was analyzed using the Mint® software (Mint® Medical GmbH, Germany). The surface areas were plotted as a function of time with baseline images, which corresponded to the non-irradiated lesions, as time point 0. The overall lesion area reduction was calculated with respect to the largest lesion area. When applicable, data were reported as mean ± standard deviation.

## Results

### Patients and Treatment Characteristics

From our database, 126 out of 168 (75%) patients were found to receive checkpoint inhibition and RT. Of these patients, 53% (67/126) were treated simultaneously, and 24 out of 67 (36%) met the inclusion criteria and were eligible for lesion analysis.

AbE was observed in 29% (7/24) of the cases as lesion shrinkage outside V10%. We analyzed 58% female and 42% male patients with a mean age of 64 ± 13 years. Fifty-four percent were diagnosed with malignant melanoma, 29% with non-small cell lung cancer, and 13 and 4% with renal cell carcinoma (RCC) and head and neck cancer (H&N), respectively. Fifty-four percent of the analyzable patients received pembrolizumab, the mean IT duration was 40 ± 28 weeks. Most of the RT courses (60%) were applied hypofractionally. Three patients were excluded from further analysis due to unreliable radiological images such as missing contrast agent in the CT, pneumonitis or atelectasis of the lung in the target lesion area. Baseline characteristics of all included patients are demonstrated in Table 1.

**Table 1:** Baseline demographics and treatment characteristics of all included patients.

**Characteristic**	**Value**
No. of patients	24
Age, years (range)	64 ± 13 (40–89)
Sex	
Male, *n* (%)	10 (42)
Female, *n* (%)	14 (58)
Primary tumor	
MM, *n* (%)	13 (54)
NSCLC, *n* (%)	7 (29)
RCC, *n* (%)	3 (13)
H&N, *n* (%)	1 (4)
IT	
Pembrolizumab, *n* (%)	13 (54)
Nivolumab, *n* (%)	11 (46)
IT duration, weeks (range)	40 ± 28 (4–115)
RT during IT	
No. of RT (range)	2 ± 1 (1–3)
CFX, *n* (%)	6 (14)
HFX, *n* (%)	25 (60)
SRS, *n* (%)	11 (26)
Analysis	
AbE, *n* (%)	7 (29)
PD, *n* (%)	5 (21)
PR, *n* (%)	5 (21)
MR with IT alone, *n* (%)	4 (17)
Image unreliable, *n* (%)	3 (13)

*Unless otherwise indicated, values represent means ± standard deviation. MM, melanoma; NSCLC, non-small cell lung cancer; RCC, renal cell carcinoma; RT, radiotherapy; CFX, normofractionated radiotherapy; SRS, stereotactic radiosurgery; HFX, hypofractionated radiotherapy; IT, immunotherapy; AbE, abscopal effect; PD, progressive disease; PR, partial response; MR, mixed response*.

The seven patients (two males and five females) exhibiting AbE had an average age of 61 ± 12 years. Three of them were diagnosed with MM, three with NSCLC, one with RCC. The MM patients received pembrolizumab, the NSCLC, and RCC patients received nivolumab with an average duration of 45 ± 35 weeks. Eighty-two percent of the RT courses were applied with high single doses as HFX (41%) or SRS (41%), and 18% normofractionated. Patients were irradiated for one (*n* = 1), two (*n* = 2), or three (*n* = 4) times with an average total EQD2 of 120.0 ± 37.7 Gy irrespectively of the number of irradiations fields and their localization. Radiotherapy was applied between 1 and 49 days (mean = 16 ± 15 days) with the first RT being performed at 19.5 ± 12.3 weeks after the induction of immunotherapy. In these patients, one to six (mean = 3 ± 2) metastatic lesions were analyzed.

Independent of the number of metastases diagnosed, each patient had only one lesion outside the irradiation field which regressed. Lesions were detected at the lung (*n* = 3), adrenal gland (*n* = 1), axillar lymph node (*n* = 1), mediastinal lymph node (*n* = 1), and at the perirenal region (*n* = 1). The AbE was observed at 20 ± 6, 5 ± 1, and 6 ± 1 weeks after the first (*n* = 2 patients), second (*n* = 3 patients) or third (*n* = 2 patients) RT, respectively, with an average lesion area reduction of 68.4 ± 23.6%. Baseline demographics of AbE patients are shown in Table 2A. A detailed description of treatment characteristics and the corresponding AbE sites are presented in Table 2B.

**Table 2A:** Baseline demographics and treatment characteristics of patients showing AbE.

**Characteristic**	**Value**
No. of patient	7
Age, years (range)	61 ± 12 (42–77)
Sex	
Male, *n* (%)	2 (29)
Female, *n* (%)	5 (71)
Primary tumor	
MM, *n* (%)	3 (43)
NSCLC, *n* (%)	3 (43)
RCC, *n* (%)	1 (14)
H&N, *n* (%)	0
IT	
Pembrolizumab, *n* (%)	3 (43)
Nivolumab, *n* (%)	4 (57)
IT duration, weeks (range)	45 ± 35 (7–115)
RT during IT	
No. of RT (range)	3 ± 1 (1–3)
CFX, *n* (%)	3 (18)
HFX, *n* (%)	7 (41)
SRS, *n* (%)	7 (41)

*Unless otherwise indicated, values represent means ± standard deviation. MM, melanoma; NSCLC, non-small cell lung cancer; RCC, renal cell carcinoma; RT, radiotherapy; CFX, normofractionated radiotherapy; SRS, stereotactic radiosurgery; HFX, hypofractionated radiotherapy; IT, immunotherapy*.

**Table 2B:** Detailed description of treatment characteristics and the corresponding abscopal effects for each patient.

**Patient**	**Primary tumor**	**IT**	**IT duration (weeks)**	**No. of RT**	**Type of RT**	**Interval between RT (weeks)**	**Irradiated sites (*n*)**	**RT dose and fractionation regime (Gy)**	**EQD2 (Gy) for α/β = 10**	**RT duration (days)**	**Time to RT after IT induction (weeks)**	**Site of analyzed metastases (*n*)**	**Site of AbE (*n*)**	**Time to AbE after RT**	**Overall lesion reduction (%)**
1	MM	Pembrolizumab	24	2	(i) SRS (ii) CFX	(i–ii) 22	(i) Brain (ii) Brain	(i) 1 × 20 Gy(ii) 50 (2) Gy	100.00	(i) 1 (ii) 25	(i) 1 (ii) 23	Lung (5), paraaortal LN (1)	Lung (1)	4 wks after 2nd RT	75
2	MM	Pembrolizumab	31	3	(i) CFX (WBRT) (iia) HFX (iib) SRS	(i–ii) 23	(i) Brain (iia) Popliteal fossa and lower leg L. (iib) Brain	(i) 40 (2) Gy (iia) 54 (3) Gy (iib) 1 × 20 Gy	148.50	(i) 25 (iia) 28 (iib) 1	(i) 1 (iia) 27 (iib) 29	Perirenal region L. (1)	Perirenal region L. (1)	6 wks after 2nd RT	100
3	MM	Pembrolizumab	24	3	(i) SRS (ii) SRS (iii) SRS	(i–ii) 15 (ii–iii) 6	(i) Brain (ii) Brain (iii) Brain	(i) 1 × 20 Gy (ii) 1 × 20 Gy (iii) 1 × 20 Gy	150.00	(i) 1 (ii) 1 (iii) 1	(i) 14 (ii) 29 (iii) 35	Lung (2)	Lung (1)	5 wks after 3rd RT	100
4	NSCLC	Nivolumab	7	3	(i) SRS (ii) HFX (iii) HFX	(i–ii) 8(ii–iii) 7	(i) Brain (ii) Femur R. (iii) Os. Sacrum and os. ischiadicum L.	(i) 3 × 9 Gy + 1 × 20 Gy (ii) 30 (3) Gy (iii) 30 (3) Gy	157.75	(i) 8 (ii) 12 (iii) 17	(i) 3 (ii) 12 (iii) 21	Suprarenal glands (2)	Suprarenal gland (1)	4 wks after 2nd RT	44
5	NSCLC	Nivolumab	104	1	CFX	–	Cervical and supravlavicular L.	54 (2) Gy	54.00	40	29	Mediastinal LN (1), hilar L. (1), axillar LN (1), intracarinal LN (1)	Axillar LN (1)	24 wks after RT	55
6	NSCLC	Nivolumab	52	3	(i) CFX (ii) HFX (iii) SRS	(i–ii) 9(ii–iii) 4	(i) Supra- and infra-clavicular lymph drainage area (ii) 3rd rib R., iliac sacral joint R., inguinal L. (iii) Occipital L.	(i) 50.4 (1.8) Gy (ii) 30 (3) Gy (iii) 1 × 20 Gy	132.08	(i) 41 (ii) 49 (iii) 1	(i) 6 (ii) 22 (iii) 33	Lung (1)	Lung (1)	7 wks after 3rd RT	56
7	RCC	Nivolumab	32	3	(i) HFX (ii) HFX (iii) HFX	(i–ii) 19(ii–iii) 20	(i) Os. Ilium L. (ii) Hip (L.+R.), os. Pubis R. (iii) Thoracic vertebra 12, Lumbar vertebra 3	(i) 36 (3) Gy (ii) 30 (3) Gy (iii) 30 (3) Gy	97.50	(i) 16 (ii) 14 (iii) 15	(i) 4 (ii) 25 (iii) 37	Mediastinal LN (2), hilar (1), pleural (1)	Mediastinal LN (1)	16 wks after 1st RT	49

*MM, malignant melanoma; NSCLC, non-small cell lung cancer; RCC, renal cell carcinoma; RT, radiotherapy; CFX, normofractionated radiotherapy; SRS, stereotactic radiosurgery; HFX, hypofractionated radiotherapy; IT, immunotherapy; AbE, abscopal effect; SD, standard deviation; L, left; R, right; LN, lymph node; wks, weeks*.

### Case Reports

#### Patient one of Table 2B

**Figure 1:**
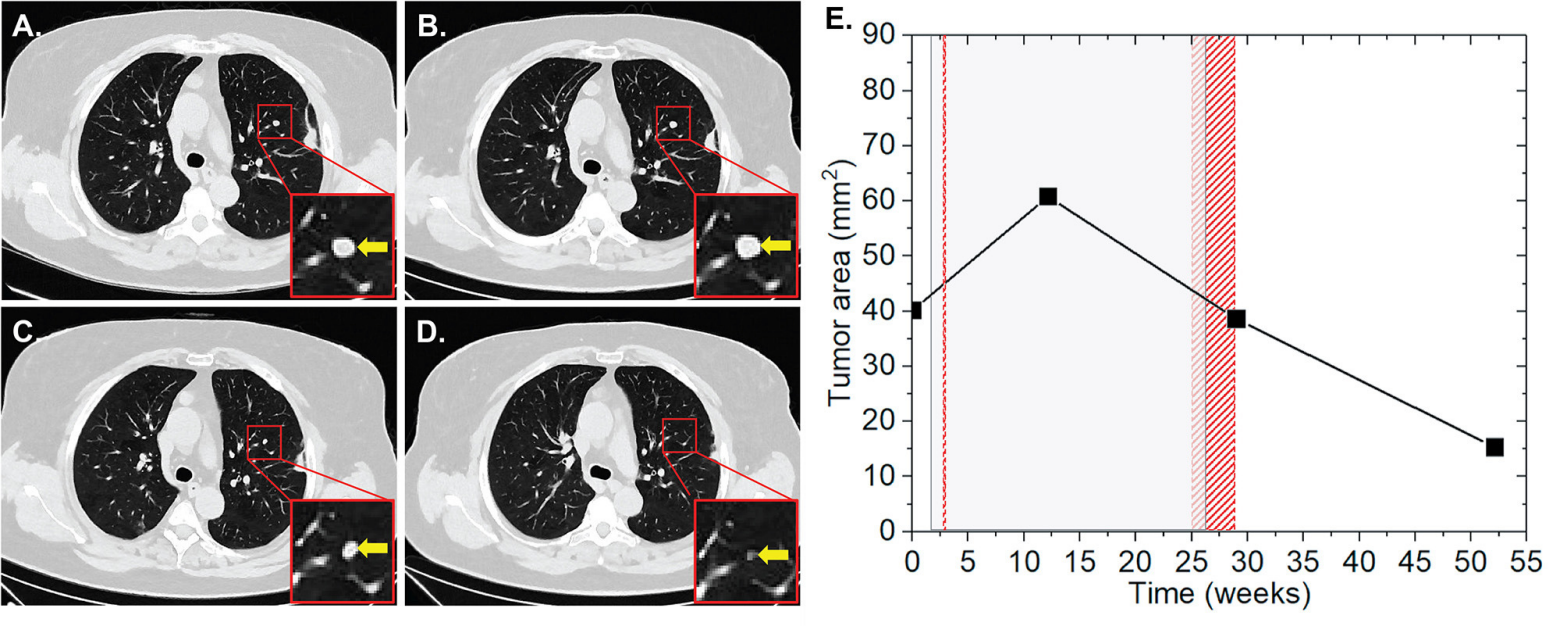
Patient 1 (Table 2B) presenting AbE in the lung. CT scans show the analyzed lesion (yellow arrows) before **(A)**, 10 **(B)**, 27 **(C)**, and 50 weeks **(D)** after the induction of pembrolizumab. **(E)** The change in the lesion surface area with respect to the administration of IT (duration = 24 weeks, gray shaded area) and concurrent RT (red lines and area) of cerebral metastases with 1 × 20 Gy (1 week after 1st IT) and 25 × 2 Gy (23 weeks after 1st IT).

In July 1998, patient one was diagnosed with an AJCC stage IIb melanoma located at the left thigh, which has been surgically resected. In May 2017, pembrolizumab was applied at 2 mg/kg for nine cycles for a period of 24 weeks due to progressive disease with metastases in the lung and brain, AJCC stage IV. During this period, the patient received two radiotherapy sessions, with a total EQD2 of 100 Gy on intracerebral lesions one (SRS) and 23 (CFX) weeks after the induction of IT. Of the six measured metastases on the CT scans, one pulmonary metastasis showed an increase in the surface area from 40.1 to 60.8 mm^2^ (52%) 10 weeks after the start of IT and 9 weeks after the first RT of cerebral metastases, applied as SRS with a single dose of 20 Gy (Figure 1). One week after the second CFX with a total dose of 50 Gy, applied with a single dose of 2 Gy, and 3 weeks after the end of IT, a regression of 37% (38.6 mm^2^) was observed, suggesting AbE. In the next CT follow-up 23 weeks later, the lung lesion continued to decrease to a size of 15.3 mm^2^, resulting in an overall lesion regression of 75%.

#### Patient two of Table 2B

**Figure 2:**
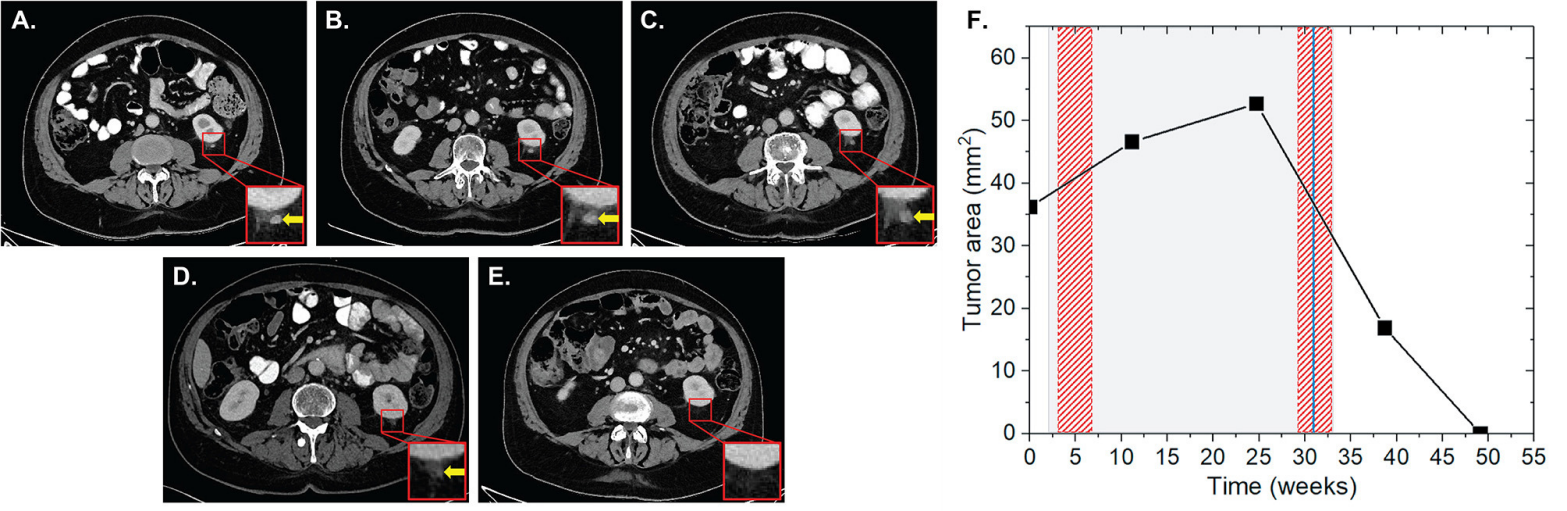
Patient 2 (Table 2B) presenting AbE a soft tissue metastasis in the perirenal region. CT scans show the analyzed lesion (yellow arrows) before **(A)**, and 10 **(B)**, 23 **(C)**, 37 **(D)**, and 47 weeks **(E)** after the induction of pembrolizumab. **(F)** The change in the lesion surface area with respect to the administration of IT (duration = 31 weeks, gray shaded area) and concurrent RT (red shaded areas) of the whole brain with 20 × 2 Gy (1 week after 1st IT) and of bone metastases in the left popliteal fossa and lower left leg 18 × 3 Gy (27 weeks after 1st IT) together with SRS of cerebral metastases with 1 × 20 Gy (29 weeks after 1st IT, blue line).

Patient two was diagnosed with an AJCC stage III malignant melanoma located at the left knee in June 2014. The melanoma was subsequently surgically removed including the lymph drainage area of the left inguinal region. In November 2015, pembrolizumab was applied at 2 mg/kg for 11 cycles for a total period of 31 weeks due to progressive disease with cerebral metastases, AJCC stage IV. During this period, the patient received two RT sessions with a total EQD2 of 148.5 Gy. The first RT was applied as normofractionated whole brain radiation therapy (WBRT) with a single dose of 2 Gy up to a total dose of 40 Gy 1 week after the induction of IT. The second RT of bone metastases of the left popliteal fossa and lower left leg was applied as HFX with a single dose of 3 Gy up to a total dose of 54 Gy at 27 weeks after the start of IT. During this RT, brain metastases were irradiated with 20 Gy in one fraction (SRS) 29 weeks after IT induction. Our analysis revealed the presence of one non-irradiated metastasis in the left perirenal area with a surface area of 36.2 mm^2^ (Figure 2). The lesion progressed to 46.6 (28.7%) and 52.7 mm^2^ (45.6%) at 10 and 23 weeks after the first application of IT, respectively, and after the first RT. In the subsequent CT scan, which corresponded to 6 weeks after the completion of IT and second RT, the lesion regressed by 67.9% to 16.9 mm^2^. Complete lesion remission was observed at 10 weeks.

#### Patient Four of Table 2B

**Figure 3:**
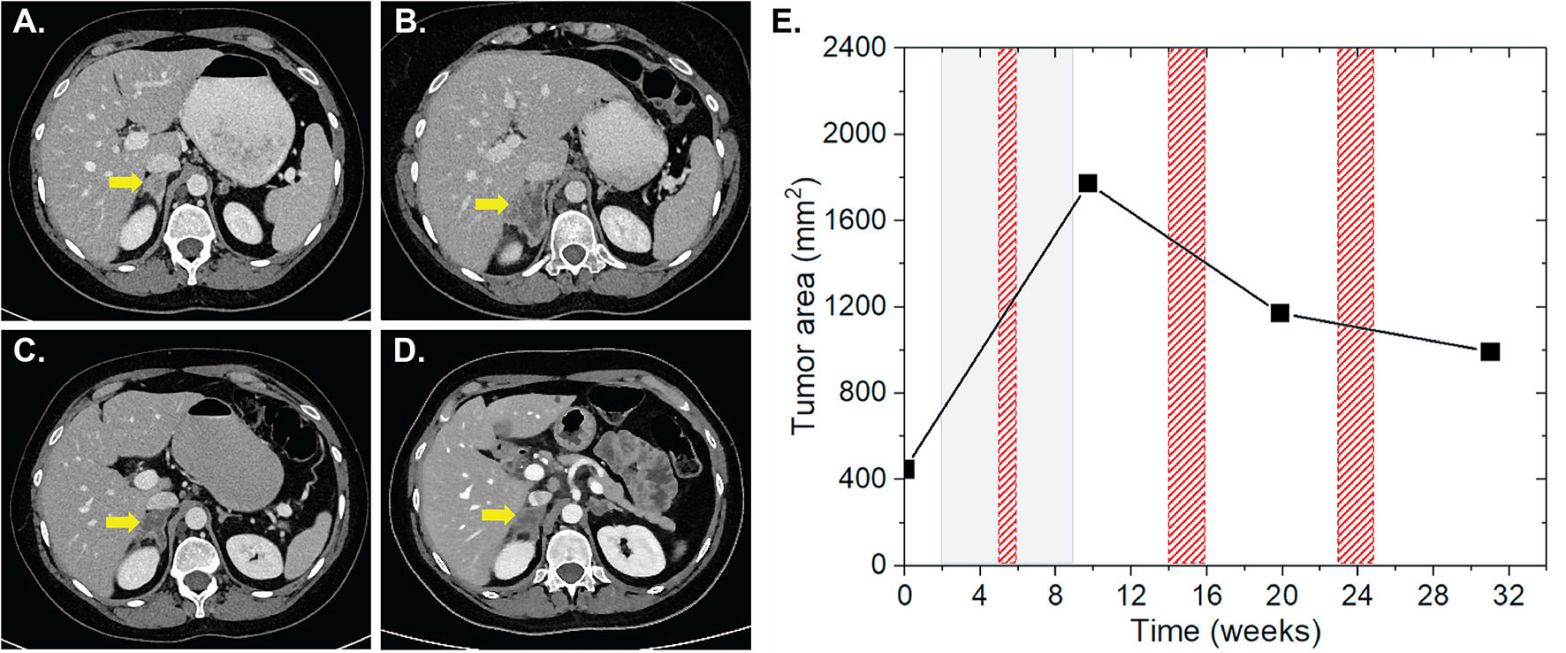
Patient 4 (Table 2B) presenting AbE in the suprarenal gland. CT images show the analyzed lesion (yellow arrows) before **(A)** and 3 **(B)**, 12 **(C)**, and 21 weeks **(D)** after the induction of nivolumab. **(E)** The change in the lesion surface area with respect to the administration of IT (duration = 7 weeks, gray shaded area) and RT (red shaded areas) of brain metastases with 3 × 9 Gy and 1 × 20 Gy (3 weeks after IT induction), and RT of bone metastases 10 × 3 Gy of the right femur, left sacrum and ischium (3, 12, and 21 weeks after induction of IT).

Patient four was diagnosed with a UICC stage IV non-small cell lung cancer (NSCLC) with metastases of the brain, suprarenal gland and bones in May 2016. The patient received a primary radiation treatment in May 2016, initially at 3 × 3 Gy on the mediastinal bulk due to superior inflow congestion. RT was then continued with a single dose of 3 Gy up to a total dose of 51 Gy. Regarding the brain metastases, SRS using the Cyberknife with 20 Gy single dose each on the 65% isodose was performed. Subsequently, the patient received palliative chemotherapy with carboplatin and abraxane. Cerebral lesions progressed in October 2016 and nivolumab was applied at 3 mg/kg for four cycles for a total of 7 weeks. Three weeks after the start of nivolumab, a concurrent stereotactic radiosurgery for cerebral metastases was applied (3 × 9 Gy and 1 × 20 Gy, each prescribed on the 65% isodose). We found non-irradiated lesions in the left and right suprarenal glands. While the left suprarenal metastasis showed a regression with IT alone, the right lesion showed an initial lesion progression from 448 to 1,773 mm^2^ at 1 and 4 weeks after completion of IT and RT, respectively, followed by 33.9% lesion regression to 1,172 mm^2^ 4 weeks after a HFX of the right femur with a single dose of 3 Gy up to a total reference dose of 30 Gy ~5 weeks after completion of nivolumab (Figure 3). During the follow-up CT scan 11 weeks later, the lesion was found to further regress to 994 mm^2^, resulting in an overall lesion regression of 44%. Three weeks after the prior CT scan the left sacrum and ischium have been irradiated with a single dose of 3 Gy up to a total dose of 30 Gy. The total EQD2 this patient received was 157.75 Gy.

## Discussion

In this study we analyzed retrospectively abscopal effects in advanced cancer patients being treated simultaneously with anti-PD1 therapy and radiation therapy. We used strict inclusion criteria for the radio-immunotherapy concept as being applied simultaneously and the radiological imaging information on distant lesions. AbEs were observed in 29% of our includable patients.

AbE was defined as radiation-induced shrinkage of distant, non-treated lesions (Mole, 1953; Andrews, 1978) and this was considered the visual evidence for the efficient immune-stimulation by irradiation. The immune system has been suggested as the key component for distant effects outside the irradiation field after local RT, defined as abscopal response. Local RT is considered to induce immunogenic cell death (ICD) associated with antigen release, cytokine production, and complement activation, leading to immune responses, and to a tumor vaccination (Formenti and Demaria, 2012; Frey et al., 2014; Barker et al., 2015). Mechanisms such as increasing the expression of the major histocompatibility complex (MHC) class I, activating dendritic cells, enhancing the presentation of tumor antigens and the migration of immune cells into the tumor micromilieu, which leads to an increase of tumor-infiltrating lymphocyte density with a broader T-cell receptor repertoire, improved effector T cell activity, and modulation of TReg cells and immune checkpoint molecule expression may contribute to improved systemic immune response after local radiotherapy (Demaria and Formenti, 2009; Formenti and Demaria, 2012).

Despite the stimulation of the immune response, RT alone does not seem to be sufficient to induce AbEs in most patients. Demaria et al. demonstrated in preclinical studies shrinkage of tumors outside the irradiation field when irradiation was combined with immunotherapy. This was naturally only observed in immunocompetent mice, indicating the indispensability of the immune system in this complex process (Demaria et al., 2004). In 2015, Reynders et al. (2015) reviewed all publications relating to the term “abscopal” in the context with RT in an oncological setting. They found that AbEs induced by RT alone are rare in the clinical and even in the preclinical setting. Interestingly, the majority of AbE cases occurred in highly immunogenic tumors such as malignant melanoma, renal cell carcinoma, and hepatocellular carcinoma (HCC) (Abuodeh et al., 2016).

Preclinical data, retrospective evaluations and case reports suggest that RT enhances the effect of IT or that radiation effects may be intensified by IT (Demaria et al., 2005; Frey et al., 2014; Ngwa et al., 2018). AbE rates of 25–52% are reported in current literature when combined treatment concepts with RT and ICIs are used (Grimaldi et al., 2014; Chandra et al., 2015). Most reports on the combination of RT and ICIs refer to patients with malignant melanoma treated with ipilimumab targeting the CTLA4 checkpoint, since it was approved for the treatment of metastatic melanoma in 2011 (Postow et al., 2012; Theurich et al., 2016). In 2014, checkpoint inhibitors targeting the PD-1 receptor were approved (pembrolizumab and nivolumab). The interaction of PD-1 and its ligand PD-L1, which may be expressed on tumor cells and antigen presenting cells, leads to a suppression of T-cell activation and thus provides an immune escape for cancer cells (Taube et al., 2012). There are many reasons why combining RT with PD-1 inhibitors might be able to provide an opportunity to boost abscopal response rates turning this rare event into a clinically relevant effect (Ngwa et al., 2018). RT can induce the expression of PD-L1 on tumor cells (Deng et al., 2014). In a study from 2016, Ribeiro Gomes et al. (2016) observed an AbE response rate of 18.7% out of 16 includable patients with solid tumors being treated with anti-PD-1 treatment and concurrent radiotherapy after disease progression occurred, all of these were diagnosed with malignant melanoma. Of all the solid tumor patients we analyzed, the 29%, which revealed AbE were either diagnosed with MM, NSLCL, or RCC, which are tumors with a high mutation frequency (Alexandrov et al., 2013).

The optimal dosing and fractionation therapy to produce the highest immunogenic benefit has not been determined yet. Single and fractionated therapy have been reported to boost AbE in combination with ICIs (Deng et al., 2014; Ngwa et al., 2018). In general, higher doses per fraction were associated with AbE. In our patient cohort, six of the seven patients showing AbE received multiple RT sessions and tended to have higher single doses. Only one patient received a normofractionated RT concept. There may be an optimal dose range where AbE is more likely to occur, or below which immune stimulation may be inferior. We assume that this range is at a high dose level (Bernstein et al., 2016).

Further questions remain about the right timing of RT and ICI application. It is difficult to distinguish between the combined effects of RIT and the effect of IT alone when applied simultaneously. We have therefore established strict inclusion criteria for the timing of radiological images.

It is also possible that patients we classified at showing AbE might in fact be presenting pseudo-progression (PsP), which is less frequent than AbE but definitely observed in analyses reporting about ICI application (Hodi et al., 2016). Evidence suggest that it could be even more frequent when being combined with RT (Trommer-Nestler et al., 2018). It is assumed that PsP is generated by attracting immune cells to the tumor by a particular mechanism like releasing neoantigens due to RT. This can lead to a larger appearance of the lesion in radiological images, but after some time the size of the lesion decreases due to treatment effect and immune response (Hodi et al., 2016). We would primarily assume that the locally irradiated tumor shows PsP during RIT but it is also thinkable that it can be observed in distant lesions. The so far reported prevalence of PsP during ICI therapy is still too low to be considered as a reliable reason for the progression observed during PD-1 blockade in the seven patients presenting AbE, but must be considered as a possible differential diagnosis.

## Conclusion

In this data analysis, we were able to show that 29% of the patients we included after applying strict inclusion criteria showed regression of lesions outside the irradiation field. We have identified AbE after radiation therapy distinctly from the treatment effects of immunotherapy alone. Most patients presenting AbE had received multiple RTs. Abscopal responses are yet rarely described in humans and systematic analyses of patients treated with radio-immunotherapy are lacking. Our results provide evidence for a clinical existence of a systemic effect of irradiation during immunotherapy and contribute to the further development of cancer therapy options, in particular with regard to combination therapies. Randomized prospective studies are required to assess whether the addition of RT to ongoing PD-1 inhibition might be able to induce reliable and durable systemic responses and provide clinical benefits. Particular attention must be paid to patient selection to find the best treatment option and clear indications when AbE induction is most likely to be effective and should be attempted. Further studies should improve the optimization of dosing regimens and the timing and sequencing of RIT concepts to determine the appropriate treatment approach for optimal and most immunogenic responses.

Our results are encouraging and represent a further step toward a possible application of RT together with ICIs in patients with advanced cancer stages to induce an AbE that enables a more efficient long-term immune response after RT.

## Ethics Statement

We include humans in this retrospective study. The study was carried out retrospectively without intervening in treatment concepts and the data evaluation is based on already existing data. The analysis was approved by the ethics committee (no. 19-1036).

## Author Contributions

MT, SYY, and CB developed the conception and design of the study. MT, SYY, TP, AB, and HG discussed the cases in interdisciplinary panels. MT acquired patient data. MT and SYY organized the database, performed all analyses, and wrote the first draft of the manuscript. SYY, TP, and AB acquired the imaging data. TP, HG, MS, ST, MB-B, JM, JH, EC, SM, and CB contributed to the manuscript. All authors contributed to the revision and read and approved the submitted version.

### Conflict of Interest Statement

The authors declare that the research was conducted in the absence of any commercial or financial relationships that could be construed as a potential conflict of interest.
