# Corrigendum: Abscopal Effects in Radio-Immunotherapy—Response Analysis of Metastatic Cancer Patients With Progressive Disease Under Anti-PD-1 Immune Checkpoint Inhibition

**DOI:** 10.3389/fphar.2019.01615

**Published:** 2020-01-31

**Authors:** Maike Trommer, Sin Yuin Yeo, Thorsten Persigehl, Anne Bunck, Holger Grüll, Max Schlaak, Sebastian Theurich, Michael von Bergwelt-Baildon, Janis Morgenthaler, Jan M. Herter, Eren Celik, Simone Marnitz, Christian Baues

**Affiliations:** ^1^ Faculty of Medicine and University Hospital Cologne, Department of Radiation Oncology and Cyberknife Center, University of Cologne, Cologne, Germany; ^2^ Faculty of Medicine and University Hospital Cologne, Radio Immune-Oncology Consortium, University of Cologne, Cologne, Germany; ^3^ Faculty of Medicine and University Hospital Cologne, Center for Integrated Oncology (CIO Köln Bonn), University of Cologne, Cologne, Germany; ^4^ Faculty of Medicine and University Hospital Cologne, Department of Diagnostic and Interventional Radiology, University of Cologne, Cologne, Germany; ^5^ Department of Dermatology and Allergology, Ludwig-Maximilians University Munich, Munich, Germany; ^6^ Department of Medicine III, University Hospital, Ludwig-Maximilians University Munich, Munich, Germany; ^7^ Gene Center, Cancer- and Immunometabolism Research Group, Ludwig-Maximilians University Munich, Munich, Germany; ^8^ Faculty of Medicine and University Hospital Cologne, Center for Molecular Medicine Cologne, University of Cologne, Cologne, Germany

**Keywords:** abscopal effect, PD-1, radio-immunotherapy, radiotherapy, combination treatment, advanced cancer disease, immune checkpoint inhibition

An author name was incorrectly spelled as “Michael von Bergwelt.” The correct spelling is “Michael von Bergwelt-Baildon”. The updated **Author Contributions** statement appears below.

“MT, SYY, and CB developed the conception and design of the study. MT, SYY, TP, AB, and HG discussed the cases in interdisciplinary panels. MT acquired patient data. MT and SYY organized the database, performed all analyses, and wrote the first draft of the manuscript. SYY, TP, and AB acquired the imaging data. TP, HG, MS, ST, MB-B, JM, JH, EC, SM, and CB contributed to the manuscript. All authors contributed to the revision and read and approved the submitted version.”

The authors apologize for this error and state that this does not change the scientific conclusions of the article in any way. The original article has been updated.

